# Is Host Metabolism the Missing Link to Improving Cancer Outcomes?

**DOI:** 10.3390/cancers12092338

**Published:** 2020-08-19

**Authors:** Christopher M. Wright, Anuradha A. Shastri, Emily Bongiorno, Ajay Palagani, Ulrich Rodeck, Nicole L. Simone

**Affiliations:** 1 Department of Radiation Oncology, Sidney Kimmel Cancer Center, Thomas Jefferson University, Philadelphia, PA 19107, USA; christopher.wright@pennmedicine.upenn.edu (C.M.W.); axs791@jefferson.edu (A.A.S.); Emily.k.bongiorno@gmail.com (E.B.); Ajay.palagani@nih.gov (A.P.); 2 Department of Radiation Oncology, University of Pennsylvania, Philadelphia, PA 19104, USA; 3 Department of Dermatology and Cutaneous Biology, Sidney Kimmel Cancer Center at Thomas Jefferson University, Philadelphia, PA 19107, USA; ulrich.rodeck@jefferson.edu

**Keywords:** host metabolism, obesity, caloric restriction, dietary interventions, cancer therapy, radiation

## Abstract

For the past 100 years, oncologists have relentlessly pursued the destruction of tumor cells by surgical, chemotherapeutic or radiation oncological means. Consistent with this focus, treatment plans are typically based on key characteristics of the tumor itself such as disease site, histology and staging based on local, regional and systemic dissemination. Precision medicine is similarly built on the premise that detailed knowledge of molecular alterations of tumor cells themselves enables better and more effective tumor cell destruction. Recently, host factors within the tumor microenvironment including the vasculature and immune systems have been recognized as modifiers of disease progression and are being targeted for therapeutic gain. In this review, we argue that—to optimize the impact of old and new treatment options—we need to take account of an epidemic that occurs independently of—but has major impact on—the development and treatment of malignant diseases. This is the rapidly increasing number of patients with excess weight and its’ attendant metabolic consequences, commonly described as metabolic syndrome. It is well established that patients with altered metabolism manifesting as obesity, metabolic syndrome and chronic inflammation have an increased incidence of cancer. Here, we focus on evidence that these patients also respond differently to cancer therapy including radiation and provide a perspective how exercise, diet or pharmacological agents may be harnessed to improve therapeutic responses in this patient population.

## 1. Introduction

The seminal work by Warburg and colleagues directed attention to the metabolic interface between tumor cells and the host organism [1]. Much of the work accomplished since has focused on how tumor cells marshal resources in their microenvironment to meet their metabolic demands. By contrast, less is known about how metabolic states of the body beyond the tumor itself affect tumor progression and therapy responses. Obesity and poor diets have been recognized as a profound contributor to the global burden of disease contributing to more than four million deaths annually [2]. While the functional contribution of obesity to high blood pressure, stroke, heart disease and type II diabetes is widely recognized, its contribution to cancer outcomes, responses to therapy and toxicity is less appreciated. In the following we will outline evidence in support of the notion that metabolic states and diseases associated with obesity impact not only cancer incidence, but also the response to treatment including radiation- and chemo- therapy. A better understanding of these relationships is urgently needed to address the consequences for cancer therapy on the dramatic increase in obesity in Western societies and, increasingly, societies around the world. We will further outline therapeutic opportunities and approaches to redress the unfavorable effects of obesity-associated metabolic diseases on tumor therapy.

## 2. Altered Metabolism Impacts Cancer Outcomes

Despite the major progress in tailored cancer therapy facilitated by precision medicine approaches, overall life expectancy in the USA has decreased for three consecutive years, representing the longest consecutive decline in American lifespan since the period between 1915 and 1918, which included World War I and the Spanish Flu pandemic [3]. In part, this may be attributed to increased cancer incidence in overweight and obese patients. Annually in the USA alone, 85,000 cancer cases are linked to obesity and this number is projected to rise to 500,000 cases by 2030 [4]. The UK has similarly experienced a marked increase in obesity-related cancer cases as documented in a landmark study of 5.2 million patients, indicating a positive linear relationship between BMI (body mass index kg/m^2^) and cancer incidence [5].This increase in obesity-related cancers is mirrored by an estimated 20% excess increase in overall cancer mortality [5] and has been noted for a broad range of malignancies including breast, lung, colon, pancreatic, esophageal, genitourinary and gynecological cancers [6,7]. Postmenopausal women with high BMI have been shown to be at a 20%–40% higher risk of developing hormone positive breast cancers [8], while obese premenopausal women are at a greater than 42% risk of developing hormone negative or triple-negative breast cancer (TNBC) [9].

Conversely, underweight patients have a lower rate of cancer incidence compared to the general population. A retrospective cohort study of women with a history of anorexia nervosa demonstrated a comparable 50% decrease in the incidence of breast cancer, suggesting that caloric restriction (CR) in humans may confer protection against invasive breast cancer [10]. Similarly, a Danish registry revealed that patients with a diagnosis of anorexia nervosa manifested a lower overall cancer risk for a variety of cancers [11].

**Figure 1:**
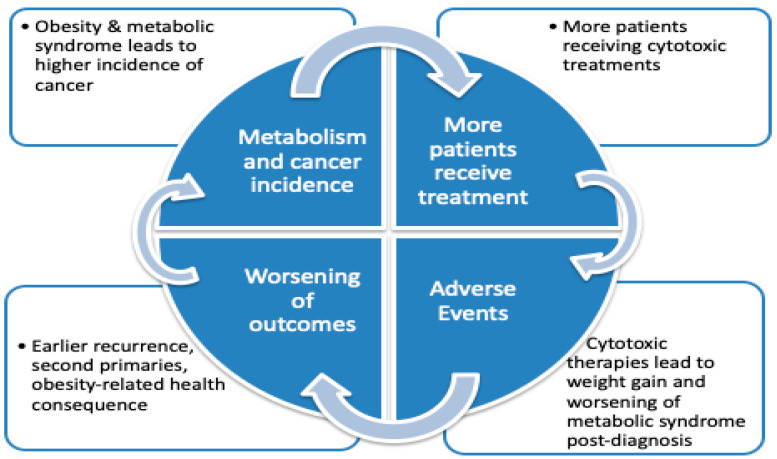
Host and tumor metabolism: a vicious cycle. Obesity, metabolic syndrome and a chronic inflammatory state have led to an increase in the incidence of obesity-related malignancies and expansion of patients undergoing chemo-and radiotherapy. These cytotoxic treatments have been linked to weight gain and development of metabolic syndrome post-diagnosis leading to poor outcomes, earlier recurrences and potential development of subsequent obesity-related cancers.

Additional evidence suggests that the relationship between increased bodyweight and cancer extends to patients who develop metabolic syndromes both during and following cancer treatment [12,13]. In fact, weight gain frequently occurs following a cancer diagnosis. Breast cancer patients gain ten to twenty pounds on average in the first year after diagnosis and this weight gain is directly linked to worse cancer outcomes. The nurses’ health study of 5204 participants with invasive, nonmetastatic breast cancer examined the impact of weight gain after time of diagnosis on clinical outcomes. An increase in BMI during treatment was associated with elevated rates of breast cancer death, distant recurrence and all-cause mortality [14]. A randomized multi-institutional phase III clinical trial enrolling breast cancer patients (women’s intervention nutrition study), revealed that reducing fat intake was associated with a significant increase in relapse-free survival than the control group [15,16]. The cause for weight gain after a cancer diagnosis is poorly understood, but appears to be multifactorial. In part, patients may reduce physical activity and increase food intake as a means to cope with their diagnosis [17,18,19]. In addition, cancer therapy adversely affects the metabolic state of the patient and contributes to weight gain. For example, chemotherapy is often administered with a pretreatment dose of steroids. Despite the relatively low doses, incorporation of steroids into a treatment regimen appears to be associated with weight gain [20,21]. Anti-estrogen therapies used in breast cancer and androgen deprivation therapies used in prostate cancer have also been shown to affect a patient’s metabolism, incite weight gain and even lead to the development of metabolic syndrome [22,23,24]. This is an integral part of a vicious circle in which therapeutic intervention is thwarted by creating and amplifying metabolic imbalances, which support cancer cell survival and growth. (Figure 1) Collectively, these observations call for the development of novel strategies to break the cycle and reset metabolic imbalances before and during cancer therapy.

### 2.1. Altered Metabolism Is Associated with Decreased Efficacy of Radiation and Other Cancer Therapies

In addition to developing cancer at higher frequency than patients with normal BMI, overweight and obese patients have worse outcomes after therapy. In part this may be due to the fact that obese patients may be undertreated due to difficulties surrounding treatment planning [25]. However, this association also holds true in patient populations receiving standard therapy. For example, prostate cancer patients with increased BMI and receiving dose-escalated intensity modulated radiation therapy (IMRT) are at increased risk of distant metastasis, prostate-specific mortality and overall mortality [26]. In a similar retrospective study in patients with breast cancer brain metastases receiving intracranial radiation, increased BMI is associated with decreased overall survival (OS) and reduced progression free survival (PFS) [27]. It has been hypothesized that a patients’ girth may be a factor in poor control, setup and reproducibility of radiation delivery to the prostate and other areas at risk. However, this seems unlikely as worse outcomes were also reported in high BMI prostate cancer patients treated with brachytherapy which allows precise delivery of radiation to the organ unencumbered by body mass [28]. Similarly, in breast cancer, it has been proposed that technical difficulties unique to obese patients cause insufficient radiation delivery to target tissues. However, based on the studies in prostate cancer it is possible that metabolic alterations associated with increased BMI contribute to radiation resistance of breast cancer cells in situ. It is tempting to speculate that the hyperinsulinemia and/or hyperglycemia prevalent in prediabetic or diabetic patients may contribute to radiation resistance of tumor cells in those patients. Furthermore, areas of high glycolytic rates are believed to confer protection from reactive oxygen species (ROS)-induced DNA damage by supplying large amounts of antioxidants [29,30]. However, studies in animal models found insufficient evidence to support a direct link between glucose uptake by cells and radiation resistance [31]. Thus, the molecular mechanisms and correlates of radioresistance of malignant cells and lesions in obese patients remain elusive.

### 2.2. Altered Metabolism Is Associated with Increased Radiation Toxicity

Across disease sites, it is clear that obesity and diabetes also increase the risk to incur adverse effects during and after radiation treatment. Breast cancer patients with a BMI ≥ 30 kg/m^2^ and undergoing radiation therapy have an increased risk of developing lymphedema and other treatment-related side effects (Analysis of the Health, Eating, Activity and Lifestyle Study). [32] In addition, overweight and obese men are more prone to developing late pelvic symptoms after radiotherapy for prostate cancer, including higher rates of rectal bleeding and nocturia [33]. Similarly, in women with endometrial cancer treated with radiation, mean BMI positively correlates with higher-grade radiation-related gynecologic and cutaneous toxicities [34]. An analysis of pulmonary tumors less than 2.5 cm from the chest wall treated with stereotactic body radiation therapy (SBRT) further found that BMI was the strongest predictor of chest wall pain after treatment, with patients whose BMI was ≥29 having nearly twice the risk of chronic chest wall pain than patients with lower BMI [35]. Additionally, patients with poor glycemic control are noted to have increased cognitive and functional decline as noted by white matter changes following whole brain radiotherapy for brain metastases [36].

## 3. Metabolic Alterations and Inflammation

Both cancer development and obesity-related comorbid conditions are closely linked to chronic inflammation. Rudolf Virchow first suggested that cancer may develop at sites of chronic inflammation based on his observation of leukocytic infiltrates in tumor tissues. A broad base of epidemiological evidence collected since supports this concept and has demonstrated that tumor cells often co-opt mediators of chronic inflammation to support malignant traits ranging from cell proliferation to metastatic spread [37]. Adipose tissue has been recognized as a rich source for autocrine, paracrine and endocrine proteins—collectively termed adipokines—that act in concert with proinflammatory cytokines to contribute to organism-wide chronic inflammatory states. The systemic effects of chronic inflammation in the fat tissue of obese individuals on tumor cell growth and development are poorly understood. However, evidence is emerging that obesity-linked inflammation has extensive effects on tumor growth, antitumor immune responses and, potentially, the efficacy of immunotherapeutic approaches [38,39,40].

The immune environment of adipose tissues in lean and overweight or obese patients is markedly different, both in cytokine expression patterns and in cell composition and phenotype. This is perhaps most evident for innate immune cells of the myeloid lineage, i.e., monocytes and macrophages that reside in fat tissue. Whereas in lean individuals these commonly display a predominant regulatory M2 phenotype, in obese individuals they are polarized toward the M1 phenotype characterized by the production of proinflammatory cytokines including tumor necrosis factor (TNF)α, interleukin (IL)-6 and IL-1β, as well as monocyte chemoattractants including monocyte chemoattractant protein (MCP)-1 and macrophage inhibitory factor (MIF) [41,42]. Within the adipose tissue these macrophages may then be biased towards the M1 phenotype by exposure to oxidative stress, hypoxia and recognition of pattern/danger associated molecular signals (PAMPS/DAMPS) that activate Toll-like receptors (TLRs) [43]. TLR stimulation triggers nuclear factor (NF)-κB-signaling and reinforces production and secretion of proinflammatory cytokines [44,45,46,47]. The inflamed status of adipose tissues in obese individuals exerts systemic effects and affects immune homeostasis at distant sites including malignant tumors. For example, serum levels of several cytokines including IL-6 and TNFα are elevated in obese individuals [48]. IL-6 activates signal transducer and activator of transcription (STAT)3-signaling in immune and tumor cells and skews the differentiation state of myeloid cells towards immunosuppressive phenotypes [49,50]. Importantly, reduced caloric intake and regular exercise have been shown to reduce the serum concentrations of IL-6, TNFα and are associated with reduced activity of tumor-promoting transcription factors, including activator protein (AP)-1, STAT3 and NF-kB in various tissues [51,52,53,54]. An emerging area of research is the role of extracellular vesicles as a mode of intercellular communication regulating inflammation, insulin sensitivity and lipid metabolism. Extracellular vesicles are produced and excreted by cells where they diffuse to neighboring or distant sites carrying proteins, lipids and nucleic acids (for example messenger (m)RNAs, transfer (t)RNAs and microRNAs), serving as a novel mechanism for intercellular communication [55,56]. Obesity has been linked with higher quantity of circulating extracellular vesicles [57,58] leading to the simulation of monocyte differentiation into active macrophages and inducing proinflammatory cytokine secretion, such as IL-1 and TNF-α [59].

**Figure 2:**
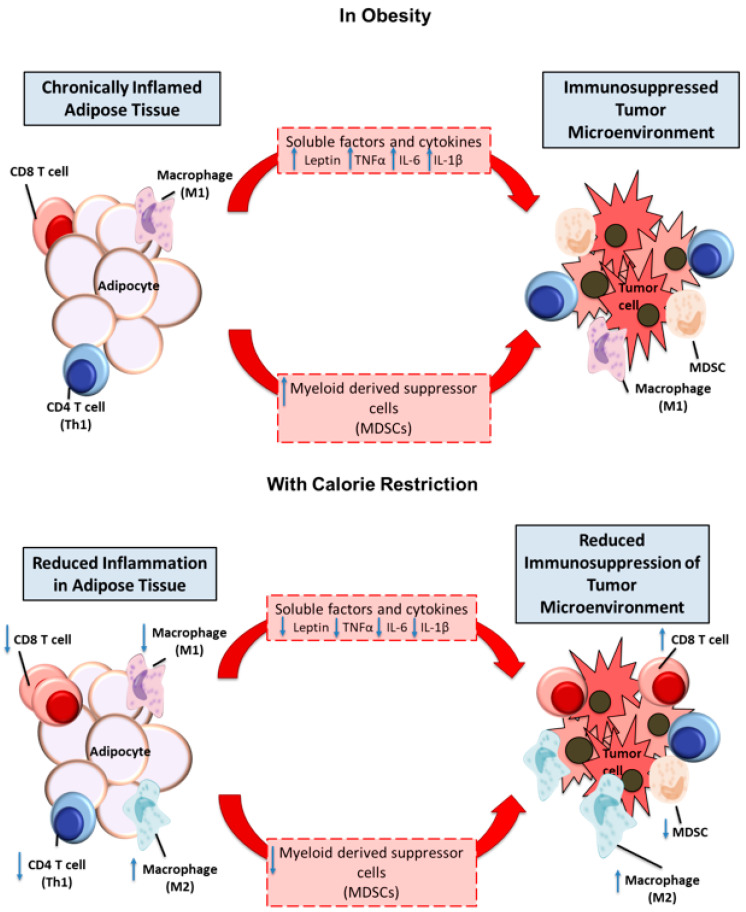
Calorie restriction contests the effects of chronic inflammation in adipose tissue on tumor development and immune responses. Adipose tissues contain activated immune cells including T lymphocytes (cluster of differentiation (CD)4, CD8) cells and proinflammatory (M1-polarized) macrophages in close proximity to adipocytes. Cytokines and adipokines secreted into the circulation, in concert with increased levels of myeloid-derived suppressor cells (MDSCs), are likely to contribute to the immunosuppressive microenvironment in tumor tissues. Calorie restriction reduces inflammation in adipose tissue by reducing T lymphocytes activity and increasing anti-inflammatory (M2-polarized) macrophages near adipocytes. Calorie restriction also reduces secreted levels of cytokines and adipokine and decreases levels of MDSC rendering the tumor microenvironment less immunosuppressive.

As mentioned above, the effects of obesity on immune homeostasis are not confined to circulating cytokines but extend to the composition and phenotype of immune cell populations in peripheral blood and tumor tissues. Perhaps most relevant to antitumor immune responses, increased levels of myeloid-derived suppressor cells (MDSCs) have been reported in the peripheral blood of obese human subjects [60]. MDSCs may serve to counteract inflammation and increase glucose tolerance [61] in obese individuals yet they have also been implicated in blunting antitumor immune responses in cancer patients through multiple mechanisms, including inhibition of tumor-infiltrating T cell proliferation and function, recruitment of regulatory T cells (Tregs) and inhibiting natural killer (NK) cell function [62]. Obesity-related modulation of NK cells through pathologically elevated levels of leptin and IL-6 has been demonstrated to reduce cytotoxicity against malignant cells [63]. Similarly, the frequencies of invariant natural killer T (iNKT) cells and mucosal associated invariant T (MAIT) cells are diminished in the setting of obesity and can be restored following weight loss [64,65,66]. In mice, higher levels of circulating dendritic cells [67] and intratumoral MDSCs [68] have been reported in tumor-bearing obese than lean animals, a phenomenon also associated with poorer responses to immunotherapy [69]. In addition to myeloid cells, other immune cell populations (for example T helper (Th)1, Th17 and Treg lymphocytes) are altered in fat tissue of obese individuals [70,71,72]. The functional significance of changes in the abundance or differentiation of these immune cells in adipose tissue to tumor development in obese individuals remains elusive. Collectively, these data reinforce the notion that excess adipose tissue has systemic effects on the innate and adaptive immune systems which, in aggregate, compromise effective antitumor immune responses. (Figure 2) Much work remains to be done to understand how changes in immune cell composition and phenotype are linked to endocrine and paracrine effects exerted by adipose tissue-derived cytokines.

## 4. Treating the Host: Diet and Pharmaceutical Intervention

**Figure 3:**
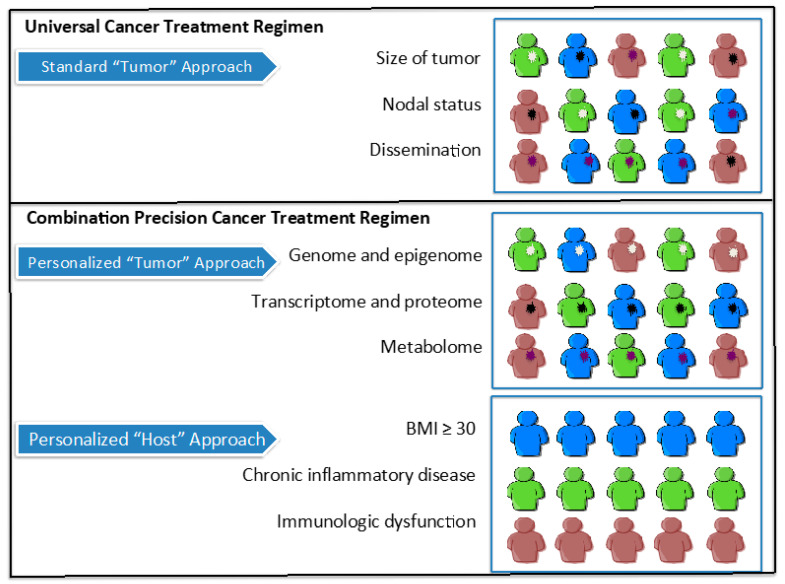
Futuristic treatment strategy: the marriage of host and tumor personalized therapies. Conventional approaches to cancer treatment regimens employ traditional classification of malignant tumors (TNM) into stages utilizing alphanumeric codes describing size of the primary tumor, nearby (regional) lymph node involvement and distant metastases. Future treatment approaches should aim to combine precision “tumor” medicine with precision “host” medicine to optimize clinical outcomes. Precision medicine attempts to personalize treatment based on molecular profiles of the tumor. This should be combined with an approach to account for distinct patient characteristics, such as body mass, disease states conferring chronic inflammation and immunologic dysfunction.

The close epidemiologic relationship between increased BMI, inflammation and cancer raises the question how best to correct and disrupt pathogenic mechanisms supporting tumor growth and treatment resistance. An obvious point of intervention is nutrition—which has effects on adipose tissue mass and the hormones and intermediaries produced by fat cells. In addition, numerous agents currently used or tested for cancer treatment affect ‘metabolic’ targets. These include inhibition of anabolic mechanisms, i.e., the insulin growth factor-1-receptor/protein kinase B (IGF1-R/PKB) axis and mammalian target of rapamycin (mTOR) and activation of catabolic mediators including adenosine monophosphate-activated protein kinase (AMPK) [73]. The IGF-1R-signaling pathway is known to play a role in the progression and metastasis of many types of cancers. The phosphorylation of the receptor tyrosine kinase domain has been shown to trigger the downstream survival pathway involved in malignant transformation. Increased expression of the components of the IGF-1R-signaling pathway including IGF-1, IGF-1R have been associated with radio and chemoresistance. Adipocytes of obese individuals produce a 2-fold increase in IGF-1 compared to normal lean individuals [74] (Figure 3).

### 4.1. Diet Improves Sensitivity to Cancer Treatments

While the benefits of dietary interventions on metabolic parameters, including improved blood glucose, insulin and triglyceride profiles, have been described for decades, recent research has revealed that dietary interventions also has direct effects on cancer cell survival and growth by increasing apoptosis and decreasing the rate of proliferation, angiogenesis, hormone levels and growth factors [75]. In fact, a systematic review of 59 studies on dietary restriction in murine cancer models reveals that the therapeutic effect of CR is broad-based and applicable across multiple tumor entities and significantly reduces the odds ratio (OR) of cancer induction (OR 0.2, 95% CI: 0.12–0.34 relative to controls) [76]. These encouraging results have motivated several clinical weight loss trials using hypocaloric diets in oncology patient populations. Preclinical evidence strongly supports the notion that nutrient restriction can be used concurrently with radiation to increase efficacy. In several models of triple negative breast cancer (4T1 and 67NR), CR has been shown to have an additive effect to decrease the primary tumor growth when combined with radiation and was noted to inhibit the IGF-1R/Akt pathway [77]. A similar effect was noted in both hormone sensitive (LNCaP) and hormone insensitive (PC3) models of prostate cancer in which CR increased the efficacy of radiation [78]. In both the breast and prostate cancer models, metastatic burden was decreased and overall survival was increased. Multiple in vivo studies in lung, colorectal, melanoma, pancreatic and breast cancer models [77,79,80] have shown that short-term or time-restricted diet protect the normal cells from toxicities due to radiation and chemotherapy. Normal cells are thought to protect themselves against the extreme changes in their microenvironment caused by starvation by adapting, temporarily shutting down growth pathways, decreasing IGF-1, increasing AMPK and using alternative sources of energy such as ketone bodies. Tumor cells are however unable to adapt to the sudden changes caused by diet, are unable to use alternate energy sources in absence of glucose and hence become more sensitive to the treatment [81]. Exposure to a calorically restricted diet has been shown to reduce systemic glucose, modulate IGF-1-signaling pathways and induce the activation of AMPK [82,83,84]. Furthermore, preclinical and human evidence has shown that CR is associated with reduced inflammatory-signaling cascades [85,86]. A multicenter trial conducted in healthy non-obese adults showed reductions in circulating inflammatory markers, including intercellular adhesion molecule (ICAM)-1, leptin, C-reactive protein and TNF-α in patients randomized to a 25% reduction in calories compared an ad lib diet [87]. CR has also been shown to enhance immunosurveillance by increasing the tumor infiltrating CD8+ T cells while reducing the regulatory T cells [88]. Ketogenic diets have also been shown to enhance radiotherapy in both lung cancer and glioblastoma multiforme models. Lung cancer xenografts (NCI-H292 and A549) were shown to be more responsive to the combination of radiation and chemotherapy in the presence of a ketogenic diet due to enhanced oxidative damage mediated by lipid peroxidation which led to decreased proliferation [89]. A ketogenic diet also enhanced radiation in a malignant glioma model (GL261), where animals receiving KetoCal had a better imaging response and freedom from tumor-recurrence compared to standard diet animals [90]. Furthermore, dietary interventions such as CR and the ketogenic diet may be utilized to induce radioprotective effects via a “differential stress resistance” where healthy cells are better able to adapt to glucose starvation than cancer cells [91].

In addition to the aforementioned macro alterations to diet, modifications of individual nutrients have been shown to alter sensitivities to anticancer therapies as well. Fructose and non-essential amino acids, such as serine, are implicated in cancer cell metabolism and their deprivation serves as a promising adjunct to anticancer therapies [92,93]. Methionine restriction has been shown to enhance therapeutic response in a variety of murine models, including sarcomas [94], gliomas [95], prostate cancers [96], breast cancers [97,98,99] and melanomas [100]. Additional nutrient targets that are active areas of research include asparagine [101], arginine [102,103], cystine [104], aspartate and glutamine [105], among others [92]. Together, these findings suggest a therapeutic potential from depriving patients of specific, individual nutrients as apart the effort to further precision medicine and optimize clinical outcomes.

### 4.2. Clinical Trials Using Dietary Alterations with Radiation

These encouraging preclinical data have spawned several clinical trials using diet coupled with radiation. (Table 1) Several trials have been accruing patients to ketogenic diet during radiation [106]. At the University of Iowa, ketogenic diet is being evaluated concurrently with radiation in three phase I trials: one for locally advanced pancreatic cancer, one for lung cancer and the last for patients with head and neck cancer. Tolerability of the diet is being assessed along with serum glucose, ketone and oxidative stress markers. Investigators from Leopoldina Hospital Schweinfurt in Germany have completed an initial analysis of six patients who underwent ketogenic diet with radiation and were noted to be compliant and lost fat mass by the completion of their intervention [107]. Our group recently finished recruiting patients that underwent a 25% reduction in calories during radiation for early stage breast cancers in the caloric restriction for oncology research trial (CaReFOR) [NCT01819233]. This pilot trial measured dietary adherence, body metrics, quality of life metrics, in addition to serum measures of inflammation including adipokines and IGF-1.

Clinicians should proceed with caution when selecting which patients may benefit from enrollment in clinical trials evaluating the coupling of dietary interventions with radiation. Malnutrition and subsequent loss of muscle mass occurs frequently following cancer therapies, particularly in patients with advanced-stage disease or head and neck malignancies and is associated with poor outcomes [114,115]. Profound nutritional deficits are also associated with decreased quality of life, decreased functional status and poor treatment compliance [116,117,118]. Clinical trials that alter a patient’s diet should involve the expertise of a dietician with regimented contact during and following cancer-directed therapies [119,120]. The era of personalized medicine incorporating both host- and tumor-specific factors represents an exciting strategy for furthering cancer treatments, but great care should be taken to enroll patients likely to benefit from dietary interventions.

### 4.3. Clinical Trials Using Dietary Alterations to Prevent Radiation Toxicity

While it is thought that dietary interventions can increase efficacy of radiation on tumor cells, it is also suspected that diet may play a protective role on normal tissues and prevent toxicity. Several studies are assessing the use of dietary alterations to prevent gastrointestinal toxicity from pelvic radiation with the Royal Marsden evaluating low- vs. high-fiber diet and the University College of London Hospitals using probiotics [121].

## 5. Pharmacological Modulation of Metabolism

Though dietary measures hold promise for our changing patient population, pharmacologic intervention combined with radiation could yield a similar benefit and may be better tolerated by the general population.

To date, pharmacologic agents that harness the beneficial effects of metabolism, are just beginning to be combined with radiation to try and increase efficacy of treatment. Metformin, a drug widely used in the management of type 2 diabetes, is one such example. This drug was first noted to be beneficial in reducing cancer mortality in epidemiological studies of both normal weight and obese patients [122,123]. Metformin affects AMP-K and mTOR-signaling and multiple preclinical studies have shown that modulation of these pathways may affect radiation sensitivity of a variety of cancer types, including prostate, breast, esophageal and lung cancers [124]. In addition, metformin targets tumor-associated macrophages (TAM) by reducing TAM M1-like polarization thereby decreasing cytokine secretion of IL-1β, TNFα and IL-10 and affecting cancer outcomes such as reducing metastases [125]. Currently, multiple clinical trials evaluating the addition of metformin to radiation across a variety of malignant primaries are ongoing.

Preclinical evaluation of other pharmacologic inhibitors of metabolism have also been performed to determine which may be combined with radiation in the clinic. In the obese state, overexpression of glucose transporters allows for an increase in available energy from the host to the tumor. In vitro inhibition of the glucose transporters Glut 1 (WZB117) and Glut 4 (ritonavir) enhanced radiosensitivity in both breast [109] and head and neck cancer [110] cells, respectively. ritonavir may also contribute to radiosensitization via inhibition of Akt-signaling [126]. Acting in a similar manner, glycolysis inhibitors and CR mimetics (CRM) have also been shown to affect energy and increase radiation sensitivity with 2-deoxy-D-glucose (2-DG) used in glioblastomas models [127] and dichloroacetic acid (PDK inhibitor) in prostate cancer models [111]. Other CRMs have also been shown to improve chemotherapy and immunotherapy response by depleting regulatory T cells and increasing infiltrating CD8^+^ T cells, including hydroxycitrate in NSCLC [128] and acarbose in renal cell carcinoma [108]. Another strategy, inhibiting fatty acid synthase with either cerulenin or orlistat has notably increased radiation sensitivity in both prostate [112] and head and neck squamous cell cancers [129], respectively. Preclinical studies using IGF-1R targeting antibodies, tyrosine kinase inhibitors and IGF-1/2 neutralizing antibodies to target the IGF-1R pathway have shown benefits in solid and hematological tumors [130]. However, even though the IGF-1R targeting antibodies AMG-479, IMC-A12 (cixutumumab) and TKI NVP-AEW541 have been shown to reduce tumor proliferation in pancreatic [113], breast cancer [131] and endometrial cancer [132,133] cells in preclinical models, they are likely ineffective as monotherapies. In summary, preclinical models have shown promise when radiation is coupled with inhibitors of metabolism.

Clinical results with other ‘metabolic regulators’ in conjunction with radiation, however, have largely been less encouraging. For example, inhibition of mTOR or PI3K, key metabolic pathways known to be regulated by nutrients, concurrently with radiation has demonstrated no discernible clinical benefit in rectal cancer [134,135], or high grade gliomas [136]. In addition to the less than ideal clinical results, it seems toxicity such as pulmonary fibrosis and infection rates may be increased with these combination therapies [136,137,138]. Further evaluation of alternative metabolic regulators is encouraged, however. A clinical trial using 2DG, an inhibitor of glucose transport and glycolysis, with radiation demonstrated improved local tumor control in glioblastomas [127]. Promising results continue to set the stage for the design and implementation of further clinical trials assessing modulation of the metabolic process as an adjunct to contemporary cancer treatment modalities.

## 6. Conclusions

Given the global epidemic of obesity, the metabolic state of the host as it relates to cancer incidence, treatment outcomes and tumor biology, deserves heightened scrutiny. Combining the pleiotropic effects of metabolically targeted interventions with contemporary cancer treatment modalities may improve local tumor control, reduce normal tissue damage and lead to better outcomes for patients. Due to the rapid development of immunotherapy, systemic effects of metabolic deregulation on antitumor immune responses are likely to be a major focus of these efforts. It is envisioned that these studies will not only reinforce the utility of current dietary approaches prior to and during cancer therapy, but also reveal whether pharmacological modifiers of metabolism will be of further benefit. Incorporation of both host- and tumor-specific factors represents an exciting strategy for furthering cancer treatments, however, clinicians should proceed with caution when selecting which patients may derive benefit from enrollment in clinical trials evaluating of dietary interventions as an adjunct to radiation. Future work into the clinical translations and mechanisms by which dietary interventions improve tumor response to radiotherapy is warranted.

## Figures and Tables

**Table 1:** Preclinical studies evaluating the impact of obesity, dietary modification and pharmacologic modulation on the response to cancer-directed therapy.

First Author	Cancer Type	Experimental Design	Results
**Obesity**			
James, B.R. et al. [67]	renal cell carcinoma	♀ BALB/c with renal cell carcinoma on chow or HFD (DIO model)	DIO mice with RCC tumors show ↑ in T cell suppressive dendritic cell. ↑ in tumor outgrowth.
James, B.R. et al. [69]	renal cell carcinoma	♀ BALB/c with renal cell carcinoma on chow or HFD (DIO model) treated with TRAIL-encoding recombinant adenovirus (Ad5-TRAIL) in combination with CpG-containing oligodeoxynucleotides (Ad5-TRAIL/CpG) of RCC.	Ad5-TRAIL/CpG unable to reduce MDSC in tumors of DIO mice. Treatment ↑ CD8 T cell in lean mice, but not DIO mice. Poor anti tumor response.
Hale, M. et al. [68]	renal cell carcinoma	♀ BALB/c with renal cell carcinoma on chow or HFD (DIO model)	DIO mice showed ↑ in accumulation of MDSC in tumors and spleens.
**Dietary Intervention**			
Saleh, A.D. et al. [77]	breast cancer	♀ BALB/c mice with orthotopic 67NR or 4T1 TNBC tumors on ADF or CR diet +/− 8 Gy dose of RT	RT caused 16% and ADF + RT 25% growth delay in 67NR tumors. RT caused 23% and ADF + RT 45% growth delay in 4T1 tumors. RT caused 23% and CR + RT 86% growth delay in 4T1 tumors. ADF and CR improved RT response in TNBC tumors
Simone, B. et al. [78]	prostate cancer	♂ nude mice with LNCaP or PC3 tumors on CR diet +/− 8 Gy dose of RT	CR+ RT caused 80% and 55% tumor growth reduction in PC3 and LNCaP tumors, respectively. CR + RT increased time to metastases. CR + RT increased apoptosis and decreased proliferation of tumor. ↑ radiosensitivity hormone responsive and hormone insensitive prostate tumors.
Bianchi, G. et al. [79]	colorectal cancer	♀ BALB/c mice with subcutaneous colorectal tumors treated with STS with/without oxaliplatin	Short term starvation (STS) augmented the response of oxaliplatin in reducing tumor growth and glucose uptake consumption in colorectal tumors
Allen, B.G. [89]	lung cancer	♀ athymic-nu/nu mice with subcutaneous A549 or H292 lung cancer xenograft treated with chow or KetoCal diet +/− 12 Gy dose of IR (fractionated) +/− carboplatin (15 mg/kg x3 doses)	KD + IR with/without carboplatin caused a significant tumor growth reduction compared to chow diet combined with same treatments. KD + IR group showed significantly increased survival compared to IR alone. KD + IR + carboplatin group also showed increased survival compared to IR+ carboplatin alone. KD + IR tumors showed ↑ 4-HNE modified proteins compared to IR alone indicating oxidative stress caused by lipid peroxidation.
Abdelwahab, M.G. et al. [90]	glioma	albino C57BL/6 mice with GL261 glioma tumors on chow or KetoCal +/− 8 Gy fractionated dose of RT	KD + IR increased efficiency of RT compared to RT alone and KD ↑ median survival. KD + IR mice showed exponential decline in tumor growth till D60. On reintroduction of chow diet at D100, no recurrence of glioblastoma observed.
Orlandella R.M. et al. [108]	renal cell carcinoma	BALB/c mice with renal carcinoma tumors treated with acarbose	Mice with tumors on acarbose showed ↑ in splenic and intra-tumoral CD8 T cells compared to control.
**Pharmacological Modulation of Metabolism**			
Zhao, F. et al. [109]	breast cancer	human breast cancer cells MDA-MB-231 and MCF-7 treated with different doses of RT 0.25, 0.50 and 0.75 Gy +/− WZB117	RT ↑ mRNA and protein expression of Glut1, ↑ glucose uptake and induces radioresistance in breast cancer cells. Inhibition of Glut1 with inhibitor WZB117 + RT ↑ sensitivity of resistant cells to RT.
Maggiorella, L. et al. [110]	Head and Neck Cancer	hep-2 cells treated with ritonavir (200 µM) or RT (5 Gy) or ritonavir + RT	Glut4 antagonist ritonavir enhanced sensitivity to RT in Hep2 cells and ↑ Hep2 cell death
Cao, W. et al. [111]	prostate cancer	prostate cancer PC3-Bcl-2 cells and PC3-Neo cells treated with IC_25_ concentration of dichloroacetate (DCA) and 2 Gy dose of RT	Prostate cancer cells pretreated with CRM DCA showed ↑ sensitivity to RT in both cell lines. ↓ cell survival in PC3-Neo cells compared to PC-3-Bcl-2 cells.
Rae, C. et al. [112]	prostate cancer	prostate cancer PC3 and LNCaP cells treated with C75 (35 µM) +/− 2 Gy	Fatty acid synthase inhibitor C75 in combination with RT caused maximum cell death and apoptosis compared to RT administered before or after C75. C75 + RT also showed ↑ in time to growth of LNCaP spheroids.
Beltran, P.J. et al. [113]	pancreatic cancer	pancreatic BxPC-3 and MiaPaCa2 treated AMG479 +/− gemcitabine♀ athymic nude mice with BxPC3 or MiaPaCa2 tumors +AMG479 +/− gemcitabine	IGF-1R inhibitor AMG479 in combination with gemcitabine ↓ viability of the pancreatic cell line in a dose dependent manner. AMG479 + gemcitabine caused tumor growth reduction compared to gemcitabine alone.

Abbreviations: HFD—high fat diet; DIO—diet induced obesity; MDSC—myeloid derived suppressor cells; TNBC—triple negative breast cancer; ADF—alternate day feeding; CR—calorie restriction; RT/IR—radiation therapy; KD—ketogenic diet; 4—HNE—4—hydroxynonenal; IC—inhibitory concentration; +/−—with or without; ↑—increased; ↓—decreased/reduced.
